# Carbon-ion beams induce production of an immune mediator protein, high mobility group box 1, at levels comparable with X-ray irradiation

**DOI:** 10.1093/jrr/rrv007

**Published:** 2015-03-09

**Authors:** Yuya Yoshimoto, Takahiro Oike, Noriyuki Okonogi, Yoshiyuki Suzuki, Ken Ando, Hiro Sato, Shin-ei Noda, Mayu Isono, Kousaku Mimura, Koji Kono, Takashi Nakano

**Affiliations:** 1 Department of Radiation Oncology, Gunma University Graduate School of Medicine, 3-39-22 Showa-machi, Maebashi, Gunma 371-8511, Japan; 2 Department of Radiation Oncology, Fukushima Medical University, 1-Hikariga-oka, Fukushima City 960-1295, Japan; 3 Gunma University Heavy Ion Medical Center, 3-39-22, Showa-machi, Maebashi, Gunma 371-8511, Japan; 4 Department of Surgery, National University of Singapore, Level 8, NUHS Tower Block, 1E Kent Ridge Road, Singapore 119228, Singapore

**Keywords:** HMGB1, carbon-ion beams, anti-tumor immunity, damage-associated molecular pattern

## Abstract

X-ray radiotherapy activates tumor antigen-specific T-cell responses, and increases in the serum levels of high mobility group box 1 (HMGB1) induced by X-ray irradiation play a pivotal role in activating anti-tumor immunity. Here, we examined whether carbon-ion beams, as well as X-rays, can induce HMGB1 release from human cancer cell lines. The study examined five human cancer cell lines: TE2, KYSE70, A549, NCI-H460 and WiDr. The proportion of cells surviving X- or carbon-ion beam irradiation was assessed in a clonogenic assay. The D_10_, the dose at which 10% of cells survive, was calculated using a linear–quadratic model. HMGB1 levels in the culture supernatants were assessed by an ELISA. The D_10_ dose for X-rays in TE2, KYSE70, A549, NCI-H460 and WiDr cells was 2.1, 6.7, 8.0, 4.8 and 7.1 Gy, respectively, whereas that for carbon-ion beams was 0.9, 2.5, 2.7, 1.8 and 3.5 Gy, respectively. X-rays and carbon-ion beams significantly increased HMGB1 levels in the culture supernatants of A549, NCI-H460 and WiDr cells at 72 h post-irradiation with a D_10_ dose. Furthermore, irradiation with X-rays or carbon-ion beams significantly increased HMGB1 levels in the culture supernatants of all five cell lines at 96 h post-irradiation. There was no significant difference in the amount of HMGB1 induced by X-rays and carbon-ion beams at any time-point (except at 96 h for NCI-H460 cells); thus we conclude that comparable levels of HMGB1 were detected after irradiation with iso-survival doses of X-rays and carbon-ion beams.

## INTRODUCTION

Radiotherapy (RT) induces anti-tumor immune responses [1–4]. RT induces DNA damage, which triggers immunogenic death of tumor cells; this process involves changes in the expression of cell surface molecules and the release of soluble mediators [1–4]. Immunogenic cell death stimulates antigen-presenting cells and activates T-cells, resulting in the expansion of antigen-specific cytotoxic T lymphocytes (CTLs) and the production of tumor-specific monoclonal antibodies [5–7]. We recently demonstrated that tumor antigen-specific T-cell responses are induced in esophageal cancer patients both during and after chemoradiotherapy [8]. It is thought that RT-induced immunity has therapeutic effects because RT is much less effective in mouse models lacking immunocompetent cells, such as CD8^+^ T-cells. This indicates that the efficacy of RT depends on host immune responses [9–11]. Clinically, two case reports describe RT-induced immunity and its therapeutic efficacy in melanoma patients [12, 13]. In both cases, the expansion of antigen-specific CTLs and the production of tumor-specific monoclonal antibodies were observed after RT. Both cases also showed an abscopal response (the regression of a metastatic tumor located at a distance from the irradiated tumor) after RT plus immunotherapy. The molecular mechanisms underlying the activation of anti-tumor immunity have also been elucidated. Immunogenic cell death has the potential to activate immune responses both *in vitro* and *in vivo*, and one of the key molecules in this process is high mobility group box 1 (HMGB1) [3–6].

HMGB1 was originally identified as a DNA-binding protein in calf thymus, and was so named because of its electrophoretic mobility. HMGB1 comprises two DNA-binding HMG-box domains (N-terminal A and central B) and an acidic C-terminal tail. It is a highly conserved chromosomal protein that acts as a DNA chaperone [14–16]. In addition, it functions as a damage-associated molecular pattern (DAMP) when released from damaged cells. During chemotherapy and RT, damaged tumor cells release HMGB1, which then stimulates dendritic cells (DCs) via toll-like receptor (TLR) 4. This immune activating pathway plays a significant role in the prognosis of breast cancer because patients carrying a *TLR4* loss-of-function allele relapse more quickly [5]. Furthermore, the amount of HMGB1 within the tumor microenvironment is positively correlated with the survival of esophageal squamous cancer patients [8], although the significance of HMGB1 is unclear; thus HMGB1 is a well-known and important molecule involved in RT-induced anti-tumor immunity.

Carbon-ion beam RT is a new modality in cancer therapy that provides better dose distribution and a stronger biological effect than X-ray RT. Several studies report favorable results for carbon-ion beam RT, particularly for the treatment of X-ray-resistant malignancies [17–19]. The reported biological advantages of carbon-ion beams are based on their high linear energy transfer (LET). High LET beams reduce cell cycle-dependent radiosensitivity; thus they are effective against tumors with a large percentage of cells in the G0 phase of the cell cycle [20] and induce cell death regardless of intratumoral oxic status [21]. In addition, carbon-ion beams induce anti-tumor immunity in murine models [22–25]; however, no studies have examined how the human immune system responds to carbon-ion beams.

RT-induced anti-tumor immunity has the potential to improve cancer treatment; however, it is unclear whether carbon-ion beams can induce immunogenic cell death. Here, we examined the ability of carbon-ion beams and X-rays to induce the *in vitro* release of HMGB1 from human cancer cell lines originating from different organs.

## MATERIALS AND METHODS

### Cell lines, culture and irradiation

The human esophageal squamous cancer cell line, TE2, was obtained from the University of Tohoku cell bank (Institute of Development, Aging and Cancer, University of Tohoku, Sendai, Japan). The human esophageal squamous cancer cell line, KYSE70, was purchased from Health Science Research Resources Bank (Osaka, Japan). The human lung adenocarcinoma cell line A549, the large cell carcinoma cell line NCI-H460, and the human colon adenocarcinoma cell line WiDr, were purchased from the American Type Culture Collection (Manassas, VA). Cells were cultured in RPMI 1640 supplemented with 5% fetal calf serum (FCS), 50 U/ml penicillin, and 2 mM L-glutamine, and grown at 37°C in 5% CO_2_/air. RPMI 1640 and FCS were purchased from Invitrogen (Carlsbad, CA), and penicillin was purchased from Sigma–Aldrich (St Louis, MO). X-irradiation was performed using a Faxitron RX-650 (100 kVp, 1.1 Gy/min; Faxitron Bioptics LLC, Tucson, AZ). Carbon-ion beam irradiation was performed at Gunma University Heavy Ion Medical Center (290 MeV/nucleon at the center of a 6-cm spread-out Bragg peak, ∼50 keV/µm).

### Colony formation assay

Cells were seeded into 60-mm dishes and allowed to attach overnight. The cells were then irradiated with X-rays or carbon-ion beams and cultured for a further 2 weeks. Dishes were then washed with saline, fixed with 100% methanol, stained with methylene blue, washed with water, and air-dried. Colonies containing more than 50 cells were then counted. The D_10_ dose, which represents the radiation dose required to reduce the surviving fraction to 10%, was calculated using a curve-fitting method based on the linear–quadratic model: SF = exp(−αD − βD^2^), where SF is the surviving fraction and D is the dose. The relative biological effectiveness (RBE) values for carbon-ion beams were calculated as the D_10_ relative to that of X-rays.

### HMGB1 ELISA

Cells were seeded at a density of 3 × 10^5^ cells per 35-mm dish and allowed to attach overnight. The cells were then irradiated with a D_10_ dose of X-rays or carbon-ion beams. The medium was removed and replaced with 3 ml of fresh culture medium. After incubation for the indicated amount of time, the culture supernatants were collected and centrifuged. The supernatants were collected again and HMGB1 concentrations were measured using an ELISA kit (Shinotest, Tokyo, Japan) according to the manufacturer's instructions. The cells were then trypsinized, collected, and stained with trypan blue (Sigma–Aldrich, St Louis, MO), and viable cells not stained with trypan blue were counted at each time-point.

### Statistical analysis

Statistical significance was estimated using one-way ANOVA and Tukey's *post hoc* test for multiple comparisons. Error bars represent the SD. A *P*-value <0.05 was considered significant.

## RESULTS

**Fig. 1.**
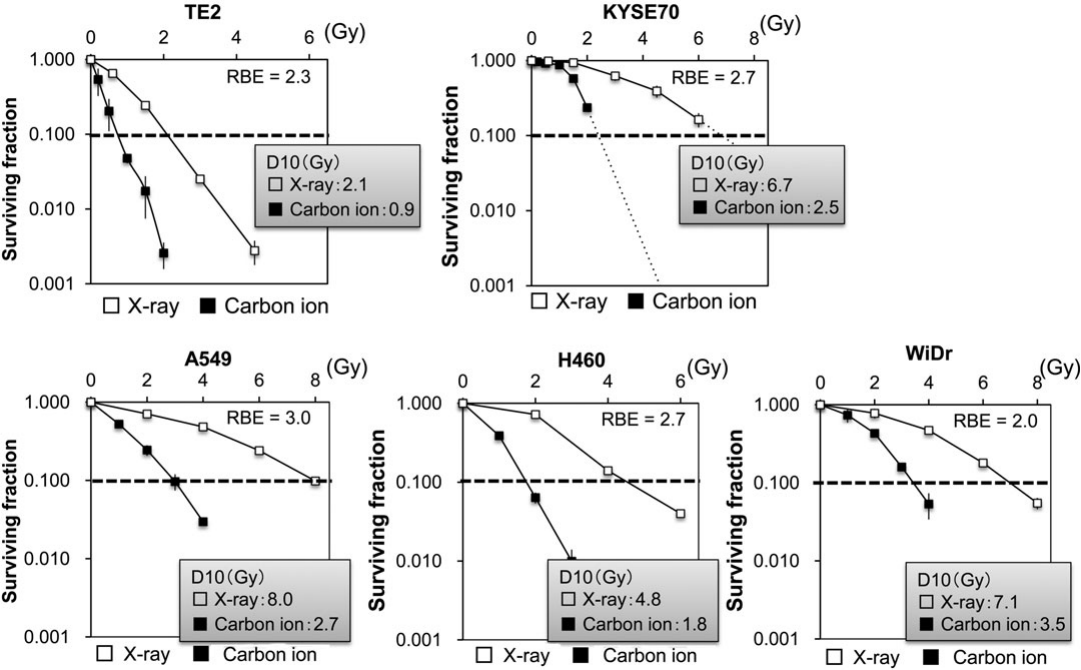
Survival curves of irradiated cancer cells. Five human cancer cell lines were treated with X-rays or carbon-ion beams at the indicated doses and then cultured for 2 weeks. The surviving fractions were calculated as a ratio relative to that of non-irradiated controls. The results are expressed as the mean ± SD of three independent experiments.

**Fig. 2.**
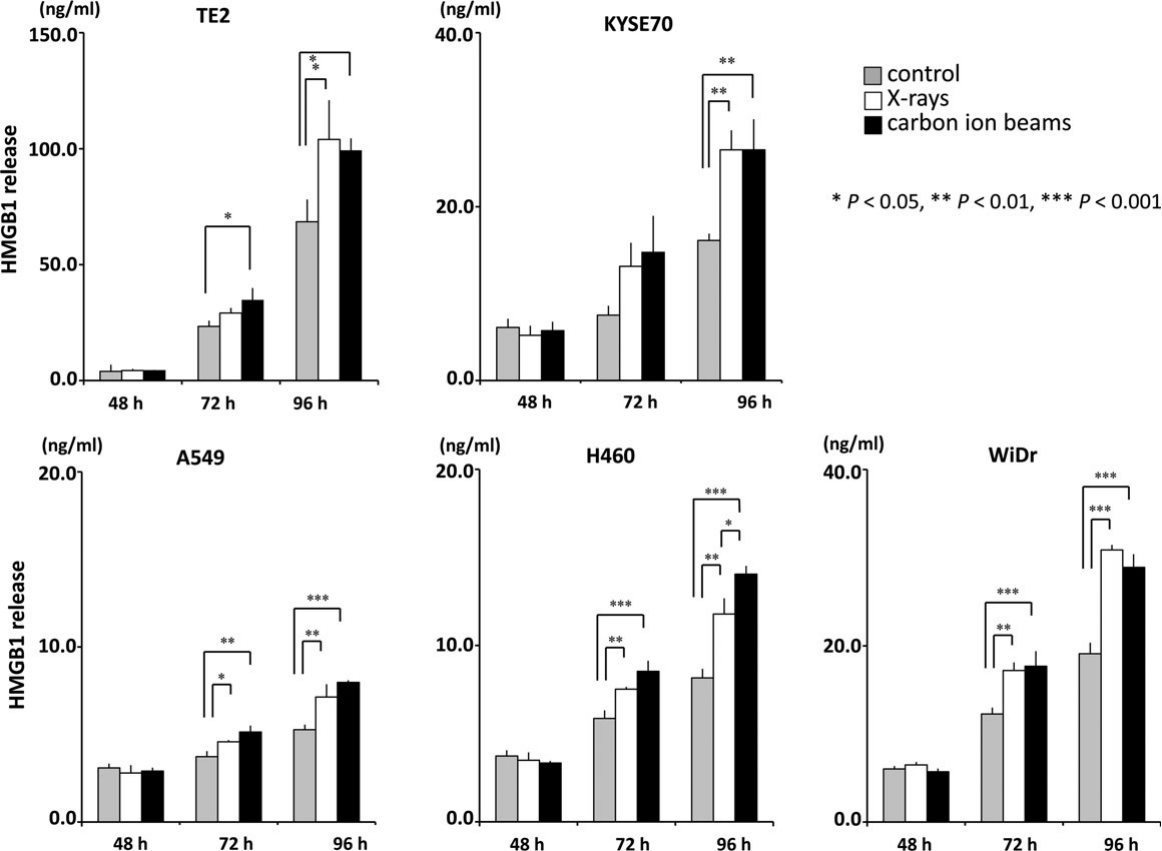
Measurement of HMGB1 release from irradiated cancer cells. Five human cancer cell lines were treated with a D_10_ dose of X-rays or carbon-ion beams and then cultured for the indicated amount of time. The concentrations of HMGB1 in the culture supernatants were measured in an ELISA. The results are expressed as the mean ± SD of three independent experiments.

**Fig. 3.**
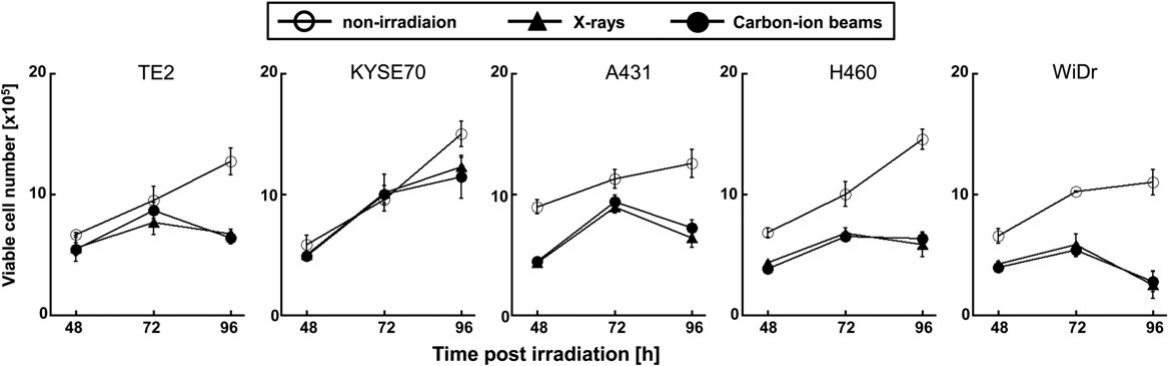
Numbers of viable irradiated cancer cells. Five human cancer cell lines were treated with a D_10_ dose of X-rays or carbon-ion beams and then cultured for the indicated amount of time. The numbers of viable cells were determined by counting cells not stained with trypan blue under a microscope. The results are expressed as the mean ± SD of three independent experiments.

To evaluate the RBE of carbon-ion beams, human cancer cell lines were treated with X-rays or carbon-ion beams, and the surviving fractions were determined in a colony-formation assay (Fig. 1). The surviving fractions decreased in a dose-dependent manner. The D_10_ X-ray doses in TE2, KYSE70, A549, NCI-H460 and WiDr cells were 2.1, 6.7, 8.0, 4.8 and 7.1 Gy, respectively, whereas those for carbon-ion beams were 0.9, 2.5, 2.7, 1.8 and 3.5 Gy, respectively. The RBE values (calculated at the D_10_ doses) for TE2, KYSE70, A549, NCI-H460 and WiDr cells were 2.3, 2.7, 3.0, 2.7 and 2.0, respectively. Next, to examine HMGB1 release, the cancer cell lines were treated with D_10_ doses of X-rays or carbon-ion beams, and the concentration of HMGB1 in the culture supernatant was measured (Fig. 2). Both X-rays and carbon-ion beams induced a significant increase in HMGB1 levels in the culture supernatants of A549, NCI-H460 and WiDr cells at 72 h post-irradiation. At 72 h post-irradiation, the level of HMGB1 was significantly increased in the culture supernatant of TE2 cells irradiated with carbon-ion beams, whereas it was not significantly increased in the culture supernatant of TE2 cells irradiated with X-rays. Furthermore, both X-rays and carbon-ion beams induced a significant increase in HMGB1 levels in the culture supernatants of all five cell lines tested at 96 h. There was no significant difference in the HMGB1 levels induced by X-rays and carbon-ion beams (except for NCI-H460 cells at 96 h, at which time-point carbon-ion beams were significantly more effective than X-rays). The D_10_ doses of X-rays and carbon-ion beams equally affected the number of viable cells of each line (Fig. 3), confirming that the D_10_ doses calculated in the clonogenic assay were equivalent in the conditions used for ELISA. In contrast to irradiated cells, non-irradiated cells continued to grow and became confluent at 96 h (data not shown), suggesting that the culture conditions caused non-irradiated cells to release HMGB1 (Fig. 2); however, the level of HMGB1 was increased significantly more in the culture supernatants of irradiated cells than in those of non-irradiated cells. Taken together, both carbon-ion beams and X-rays induced the release of HMGB1 from different human cancer cell lines, and both types of radiation were equally effective at inducing HMGB1 release when given at iso-survival doses.

## DISCUSSION

The present study examined the levels of HMGB1 in the culture supernatants of human cancer cell lines treated with X-rays or carbon-ion beams. The results showed that X-rays and carbon-ion beams significantly increased HMGB1 levels in the culture supernatants of A549, NCI-H460 and WiDr cells at 72 h post-irradiation with a D_10_ dose. Furthermore, irradiation with X-rays or carbon-ion beams significantly increased HMGB1 levels in the culture supernatants of all five cell lines at 96 h post-irradiation. There was no significant difference in the amount of HMGB1 induced by X-rays and carbon-ion beams at any time-point (except at 96 h for NCI-H460 cells); thus we conclude that comparable levels of HMGB1 were detected after irradiation with iso-survival doses of X-rays and carbon-ion beams.

Several studies have reported the release of HMGB1 from cancer cells treated with ionizing irradiation or chemotherapeutic agents. Apetoh *et al.* reported that anthracyclins and X-rays induce the release of HMGB1 from murine tumor cells [5], and Guerriero *et al.* reported that DNA alkylating agents induce the release of HMGB1 from murine tumor cells both *in vitro* and *in vivo* [26]. From a more clinical perspective, Frey *et al.* reported that chemotherapeutic agents such as 5-FU, oxaliplatin, and irinotecan (which are used for adjuvant chemotherapy for colorectal cancer), alone or in combination with X-irradiation, induce the release of HMGB1 from a colon adenocarcinoma cell line, SW480, *in vitro* [27]. Our previous study [8] showed that either a combination of chemotherapeutic agents (5-FU, CDDP and docetaxel) or X-rays induces the release of HMGB1 from human esophageal cancer cell lines. In this case, the amount of HMGB1 released by each of the cell lines varied, and X-rays tended to induce more HMGB1 release than chemotherapeutic drugs. The present study is the first to demonstrate that human cancer cells irradiated with carbon-ion beams release HMGB1. The results show that carbon-ion beams are at least as effective as X-rays at inducing HGMB1 release from tumor cells.

The amount of HMGB1 released from irradiated cells increases in a dose-dependent manner. Gameiro *et al.* reported that 100 Gy of γ-irradiation induced more HMGB1 release from cultured cancer cell lines than 10 Gy [28]. Here, we used D_10_ doses of radiation (2.1–8.0 Gy for X-rays and 0.9–3.5 Gy for carbon-ion beams), which are comparable with actual clinical doses [29, 30]. Taken together, these results suggest that carbon-ion beams should induce HMGB1 release in a clinical setting.

When released from cells, HMGB1 acts as a DAMP and plays an important role in activating acquired immunity by binding to TLR4 on DCs [5, 6]. Studies in mouse models show that cyclophosphamide is less effective against HMGB1-negative tumors [26], and that human breast cancer patients carrying a *TLR4* loss-of-function allele have a poor prognosis [5]. It is clear that intact anti-tumor immune responses are required if anti-cancer chemotherapeutic drugs and X-ray irradiation are to be effective [1–9]; indeed, this concept has been tested by combining these ‘traditional’ therapies with ‘modern’ immune-modifying therapies in a clinical setting [31, 32]. The results of the present study suggest that carbon-ion beam therapy should also be tested in combination with immunotherapy.

Several reports show that increased HMGB1 levels are negatively correlated with prognosis [33–35]. Although the details are unclear, it is possible that HMGB1 augments inflammatory reactions, resulting in the activation of cells that suppress the activity of immune effector cells. The superior dose distribution and strong DNA-damaging effects of carbon-ion beams mean that it may be possible to induce HMGB1 release only within the tumor microenvironment. This would be a new and attractive approach that cannot be used with conventional treatments. The ‘ideal’ scenario is a combination of targeted immunotherapy (which acts not only on the primary tumor site but also on distant metastasis) and carbon-ion beam therapy. This is because many patients eventually succumb to distant metastases even though local control is much improved [19].

In conclusion, the results presented herein show that the levels of HMGB1 detected after irradiation with iso-survival doses of X-rays and carbon-ion beams were comparable. Therefore, carbon-ion beams are as effective as X-rays at inducing HMGB1 release from different types of human cancer cells.

## FUNDING

This work was supported by JSPS KAKENHI Grant Number 24591834. Funding to pay the Open Access publication charges for this article was provided by JSPS KAKENHI Grant Number 24591834.
